# Flexural Strength, Elastic Modulus and Remineralizing Abilities of Bioactive Resin-Based Dental Sealants

**DOI:** 10.3390/polym14010061

**Published:** 2021-12-24

**Authors:** Maria Salem Ibrahim, Mana’a S. Alabbas, Khalid U. Alsomaly, Abdullah A. AlMansour, Alhareth Abdulaziz Aljouie, Majed M. Alzahrani, Ahmed A. Asseri, Jehan AlHumaid

**Affiliations:** 1 Department of Preventive Dental Sciences, College of Dentistry, Imam Abdulrahman Bin Faisal University, Dammam 34212, Saudi Arabia; jaalhumaid@iau.edu.sa; 2 College of Dentistry, Imam Abdulrahman Bin Faisal University, Dammam 34212, Saudi Arabia; 2160006146@iau.edu.sa (M.S.A.); 2160000413@iau.edu.sa (K.U.A.); 2160005417@iau.edu.sa (A.A.A.); 2160003472@iau.edu.sa (M.M.A.); 2160003990@iau.edu.sa (A.A.A.); 3 College of Dentistry, Qassim University, Buraydah 52571, Saudi Arabia; 361110314@qu.edu.sa

**Keywords:** sealants, bioactive, dental resin, remineralization, demineralization

## Abstract

Objective: To assess the remineralizing abilities and compare the flexural strength and elastic modulus of different bioactive pit and fissure sealants. Materials and Methods: Human enamel samples were randomly and blindly sealed with one of the following bioactive materials: BioCoat (Bc), ACTIVA KIDS (Av) and BeautiSealant (Bu). Seal-it (Si) was used as a non-bioactive sealant beside a control blank (B) group with no sealant. The sealed samples were subjected to a pH-cycling model (7 days of demineralization–remineralization cycles). The enamel surface hardness change (SHC), scanning electron microscopy-energy dispersive X-ray spectroscopy (SEM-EDX) and polarized light microscopy were used to assess the remineralizing abilities of the studied sealants. Flexural strength and elastic modulus were also assessed following the ISO 4049 protocols. One-way analysis of variance (ANOVA) was used to analyze the results. Results: Bc sealant showed the highest FS and EM (*p* < 0.05). The contact with Bc and Bu sealants showed significantly lower %SHL (*p* < 0.05) in comparison to the other. These findings were supported by the results of SEM-EDX and polarized imaging by showing higher percentages of calcium and phosphate ions with the former sealants and thinner demineralized enamel bands. Conclusion: In this study, Bc showed the highest flexural strength. Bc and Bu sealants outperformed the other studied sealants in terms of their remineralization abilities.

## 1. Introduction

Dental caries is a worldwide multifactorial infectious disease that affects the teeth in the oral cavity, leading to localized structure deterioration. It is considered one of the most serious public health issues in the field of dentistry [1]. Caries is classified as a “complex” disease with unknown biological mechanisms in its origin. It is caused by a prolonged period of imbalance in physiological equilibrium between teeth minerals and the biofilm fluids existing in the oral cavity [2]. If the dental structure started to demineralize (lose minerals), and the demineralization process was not reversed by remineralization (gaining minerals), caries can proceed and continue progressing. In such cases, treatment should be provided, which may indicate restoring the carious teeth. Low fluoride levels in drinking water and food, living in a low-income family and poor oral hygiene are all risk factors for caries [3,4].

Recent reviews showed a decline in caries prevalence in developed countries [5,6]. On the other hand, other reviews showed an increase in caries prevalence in developing countries [6,7,8]. Pit and fissure sealants were first proposed in the 1960s as a preventive measure for dental caries on the chewing surfaces of teeth [9,10]. It acts as a physical barrier on teeth surfaces to prevent food accumulation and inhibit the bacterial growth and progression of caries [9]. Various pit and fissure sealants have been utilized to prevent caries, including glass ionomer sealants [11,12,13], resin-modified glass ionomer sealants [14] and fluoride-releasing and non-fluoride releasing resin-based sealants [12,13,15].

Over the last decade, glass ionomer cements have been recognized for their many attractive clinical characteristics, as they have been used in the prevention of dental caries in pits and fissures [16]. However, glass ionomer materials have some disadvantages such as poor hydrolytic stability, low flexure and toughness and, most importantly, the short-term release of fluoride [17,18]. The probable cause that has been believed to be the reason behind the rapid decrease of fluoride release is mainly due to the initial burst of fluoride release from the glass particles themselves as they dissolve and partially vanish in the polyalkeonate acid during the setting reaction stage [19]. Moreover, the slow release that follows later on has been believed to occur as a result of the glass dissolving in the acidified water that is found on the hydrogel matrix [19].

Incorporating fluoride and other ions such as calcium and phosphate into pit and fissure sealants appears to be useful in reducing and preventing dental caries [20,21,22]. To overcome the drawbacks of the conventional materials and to enhance the performance of such preventive procedures, researchers have experimented with new bioactive resin-based materials that may play a role in the healing process of the affected dental structures [23,24,25,26]. Dental resin-based materials are polymeric materials that have been used as preventive or restorative materials in the field of dentistry. Adding remineralizing agents or particles such as nano-amorphous calcium phosphate, tricalcium phosphate or bioactive glass particles to these resin-based materials could provide an action of acid neutralization and ions releasing abilities that may be effectively utilized in caries prevention [21,24,25,27,28]. These bioactive materials can intervene and stop the progression of carious lesions and allow the damaged tissues to heal when utilized in an early stage of the disease. Such materials have the ability to release, absorb and re-release calcium, phosphate and fluoride, which will act as a reservoir of ions when the demineralization process initiates and can reverse it [12,21,29,30,31,32].

Previous studies used various methodologies to investigate the remineralization potentials of the pit and fissure sealants, including qualitative assessments such as scanning electron microscopy and quantitative assessments such as microhardness tests [27,33]. Besides assessing the remineralization abilities of dental sealants, flexural strength and elastic modulus are important to the same extent as these materials are subjected to chewing loads [34]. Recently, various bioactive materials have been introduced to the dental market to compensate for the drawbacks of the existing conventional non-bioactive materials.

However, studies have been actively developing and investigating new bioactive materials and their anti-cariogenic effects and mechanical properties [22,23,29,35,36]. Only a few studies have studied the performance of recently introduced bioactive resin-based sealants. Therefore, this laboratory study aimed to assess some of the mechanical properties and remineralizing abilities of different bioactive resin-based sealants which have recently been introduced to the dental market.

## 2. Materials and Methods

This study was designed as a blind randomized laboratory study. The study was approved by Imam Abdulrahman Bin Faisal University Institutional Review Board (IRB-2021-02-484).

### 2.1. Study Groups

The studied sealants and their compositions are presented in Table 1.

### 2.2. Mechanical Assessments: Flexural Strength and Elastic Modulus

Following the ISO 4049 protocol, samples (*n* = 9) were fabricated in stainless steel mold (2 mm × 2 mm × 25 mm) [37]. A glass slap and polyester strip were placed below the mold then the sealant was injected into that mold, another polyester strip was placed on the top and pressed using a glass slap to eliminate air bubbles [21]. The sealant material was light-cured from both sides for 20 s each using a light-emitting source (Satelec Mini LED Curing Light 1250 mW/cm^2^, A-dec Inc., Newberg, OR, USA). The specimens were dry stored in the incubator (Heraeus–Thermoscintefic, Glasgow, UK) at 37 °C for 24 h. Three-point flexure with 18 mm span (INSTRON 5965 load frame, Boston, MA, USA) was used to measure the flexural strength and elastic modulus, at a crosshead-speed of 1 mm/min on a computer controlled universal testing machine.

Flexural strength and elastic modulus were calculated using the following formula:

(1) $$F=\frac{3\mathrm{LS}}{2{\mathrm{WH}}^{2}}$$

whereby F = flexural strength, L = maximum load, S = span, W = width of the specimen and H = height.

(2) $$M=\frac{{\mathrm{LS}}^{3}3}{4{\mathrm{WH}}^{3}3d}$$

whereby M = elastic modulus, L = maximum load, S = span, W = width of the specimen, H = height and d = defluxion corresponding to the load (L). 

### 2.3. Remineralization Abilities Assessments

**Figure 1:**
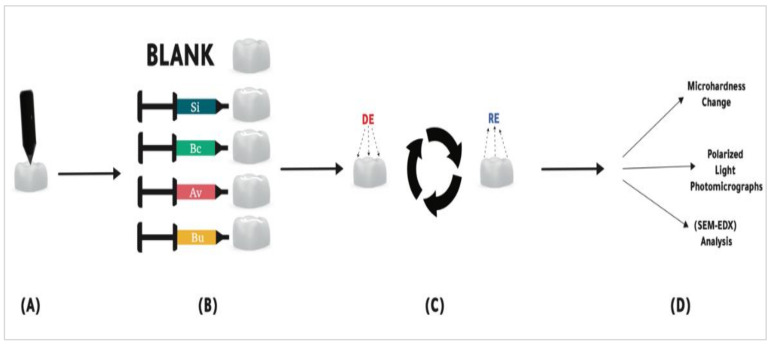
A diagram illustrating the remineralization abilities assessments’ experimental design. (**A**) The baseline Knoop hardness test of enamel samples. (**B**) The samples were sealed with different commercially available sealants and one group was left as the blank group. (**C**) The samples were subjected to pH-cycling for 7 days. (**D**) Final hardness measurements polarized light photomicrography and SEM-EDX analysis were completed.

A diagram illustrating the remineralization abilities assessments’ experimental design is presented in Figure 1.

#### 2.3.1. Sample Preparation

Recently extracted human teeth collected and cleaned with ethanol, sound enamel parts have been sectioned from the crown using a separating disk and mounted in a cylindrical acrylic block.

The acrylic base has been flattened then the enamel side was wet and serially polished using a polishing machine (BUEHLER MetaServ 250 Grinder–Polisher with Vector Power Head, Hong Kong, China) with silicon carbide sandpapers 320-Grit, 500-Grit and 1200-Grit.

The baseline surface microhardness was measured using a microhardness tester (BUEHLER MicroMet 6040 Hardness Tester, Shanghai, China) by averaging five 100 μm spaced indentations under a diamond Knoop head with 25-g force for 10 s. Samples with an average microhardness of 437 KNH ± 20% were included in the study then blindly and randomly distributed among 5 groups according to sealant materials: BioCoat (Bc), ACTIVA KIDS (Av), Seal-it (Si), BeautiSealant (Bu) and a blank (negative control) group. Each sealant was applied on the enamel and light-cured for 20 s using a light-emitting source (Satelec Mini LED Curing Light 1250 mW/cm^2^, A-dec Inc., Newberg, OR, USA).

#### 2.3.2. pH-Cycling Model

A pH-cycling model was used to mimic the loss and gain of minerals in the oral environment [27,38,39]. The samples were alternated between the demineralization cycle and remineralization cycle for 7 days following earlier studies [27,40]. Samples were air-dried then immersed in 30 mL (per sample) demineralization solution (DE): 2.0 mmol/L calcium (Ca(NO_3_)_2_·4H_2_O), 2.0 mmol/L phosphate (NaH_2_PO_4_·2H_2_O), 0.075 mmol/L acetate buffer and 0.02 ppm F at pH 4 for 6 h at room temperature. Samples were taken out from DE solution then rinsed with distilled water, dried and immersed in 15 mL (per sample) remineralization solution (RE): 1.5 mmol/L calcium (Ca(NO_3_)_2_·4H_2_O), 0.9 mmol/L phosphate (NaH_2_PO_4_·2H_2_O), 150 mmol/L potassium chloride, 0.03 ppm fluoride standard and 0.1 mol/L tris buffer at pH 7 for 18 h at room temperature.

After 7 days of pH-cycling, sealant material was carefully removed from the surface of the enamel samples then the following assessments were run.

#### 2.3.3. Surface Hardness Change

Final microhardness readings (*n* = 4 samples × 5 readings) were obtained using the same parameters as the baseline measurements. Surface hardness change was measured using the following formula [27]:

(3) $$\%\mathrm{SHC}\;=\;\frac{\mathrm{Surface}\;\mathrm{Hardness}\;\mathrm{after}\;\mathrm{pH}\;\mathrm{cycling}\;-\;\mathrm{Baseline}\;\mathrm{Surface}\;\mathrm{Hardness}}{\mathrm{Baseline}\;\mathrm{Surface}\;\mathrm{Hardness}}\;\times \;100$$

#### 2.3.4. Scanning Electron Microscopy with Energy-Dispersive X-ray Spectrometer (SEM-EDX) Analysis

Two samples from each group were gold coated (Q150T Plus Turbomolecular pumped coater Quorum, Lincolnshire, UK). Four areas from each sample were randomly selected for SEM-EDX analysis. Standardized high-resolution spectra of the elemental distributions on the surface enamel have been obtained, expressed in weight percent, then calculated using the backscattered electron collector attached to a scanning electron microscope (JSM-6610 Series Scanning Electron Microscope JEOL). The data were acquired and analyzed using (Aztec 4.1 software NanoAnalysis Oxford Instruments, London, UK). Based on weight percentages, spectra of oxygen (O), calcium (Ca), phosphate (P) and fluoride (F) were normalized.

#### 2.3.5. Polarized Light Photomicrographs

Two samples from each group were sectioned longitudinally using (IsoMet^®^ 5000 Linear Precision Saw BUEHLER, Lake Bluff, IL, USA) and positioned on a glass microscope slide for imaging. A qualitative analysis using polarized light microscopy of enamel caries lesions was run using (BX63 Motorised Research Microscope Olympus, Tokyo, Japan). The surface demineralization band under each sealant was evaluated and imaged (ECLIPSE Ti, Nikon, Tokyo, Japan) and (cellSens V2., Olympus, Tokyo, Japan).

### 2.4. Statistical Analysis

One-way analysis of variance (ANOVA) was used to analyze the results. Multiple comparisons between the studied groups were conducted using Bonferroni’s multiple comparison tests. All the statistical analyses were performed by Stata/IC 14.2 (Stata, College Station, TX, USA) at an alpha of 0.05.

## 3. Results

### 3.1. Flexural Strength and Elastic Modulus

**Figure 2:**
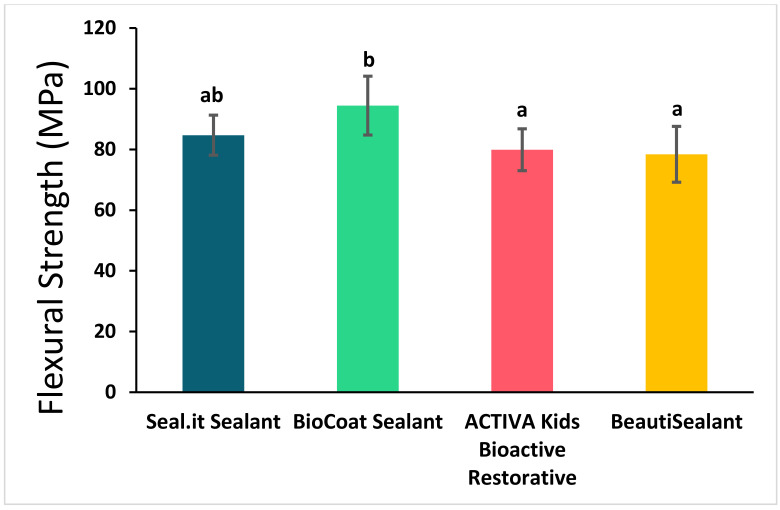
Flexural strength of the studied sealants (mean ± SD). Values indicated by different letters (a,b) are statistically significant and different from each other (*p* < 0.05).

**Figure 3:**
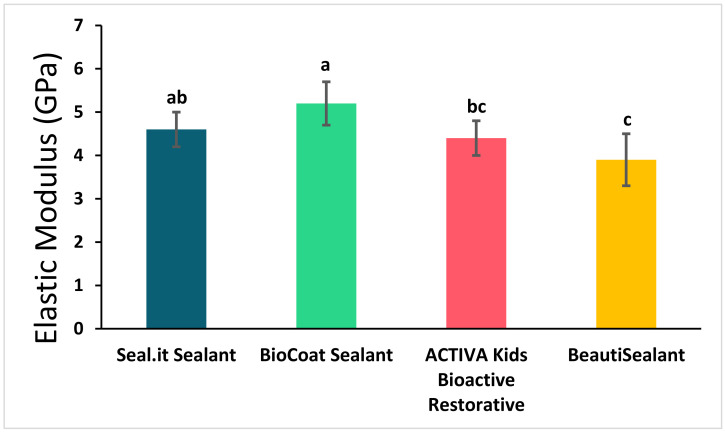
Elastic modulus of the studied sealants (mean ± SD). Values indicated by different letters (a–c) are statistically significant and different from each other (*p* < 0.05).

The flexural strength and elastic modulus of the studied sealants are represented in Figure 2 and Figure 3 and Table 2. Bc significantly showed the highest flexural strength (94.4 ± 9.7 MPa) and elastic modulus (5.2 ± 0.5 GPa) compared to the other bioactive sealants; Av and Bu (flexural strength: 75.0 ± 14.4 MPa and 75.6 ± 12.0 MPa, respectively, and elastic modulus: 4.1 ± 0.8 GPa and 3.8 ± 0.8 GPa, respectively) (*p* < 0.05). However, the non-bioactive sealant (Si) was not statistically or significantly different from the three bioactive sealants in flexural strength and from Bc and Av in elastic modulus (*p* > 0.05).

### 3.2. Surface Hardness Change

The surface hardness change of the studied sealants after 7 days in the pH-cycling model is represented in Table 2. The enamel samples with no sealant applied (negative control) showed the highest surface hardness loss followed by the Si group. Av bioactive sealant showed lower enamel hardness loss in comparison to both control groups. However, this difference was not statistically significant (*p* > 0.05). Bc and Bu showed surface hardness gain, which were significantly different from the other three groups (*p* < 0.05).

### 3.3. Scanning Electron Microscopy with Energy-Dispersive X-ray Spectrometer (SEM-EDX) Analysis

**Figure 4:**
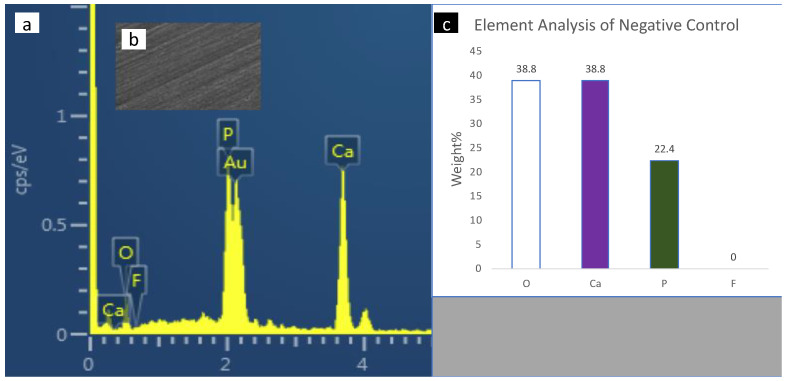
The SEM-EDX evaluation of the enamel samples (blank group): (**a**) the SEM-EDX spectrum shows the dominant elements; (**b**) SEM image of the area for which the EDX spectrum was acquired; (**c**) concentrations of elements in weight (weight percent) of the oxygen, calcium, phosphate and fluoride elements found in enamel surface under the sealants.

**Figure 5:**
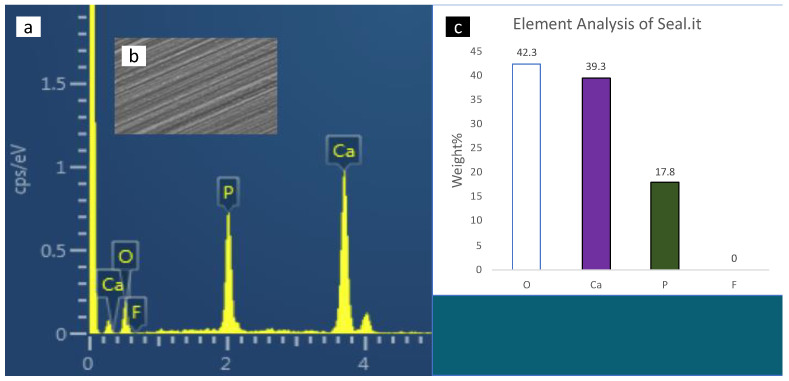
The SEM-EDX evaluation of the enamel samples under Seal-it: (**a**) the SEM-EDX spectrum shows the dominant elements; (**b**) SEM image of the area for which the EDX spectrum was acquired; (**c**) concentrations of elements in weight (weight percent) of the oxygen, calcium, phosphate and fluoride elements found in enamel surface under the sealants.

**Figure 6:**
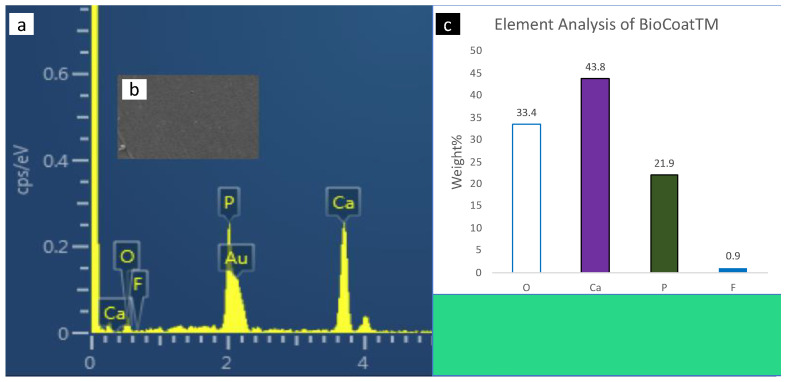
The SEM-EDX evaluation of the enamel samples under BioCoat: (**a**) the SEM-EDX spectrum shows the dominant elements; (**b**) SEM image of the area for which the EDX spectrum was acquired; (**c**) concentrations of elements in weight (weight percent) of the oxygen, calcium, phosphate and fluoride elements found in enamel surface under the sealants.

**Figure 7:**
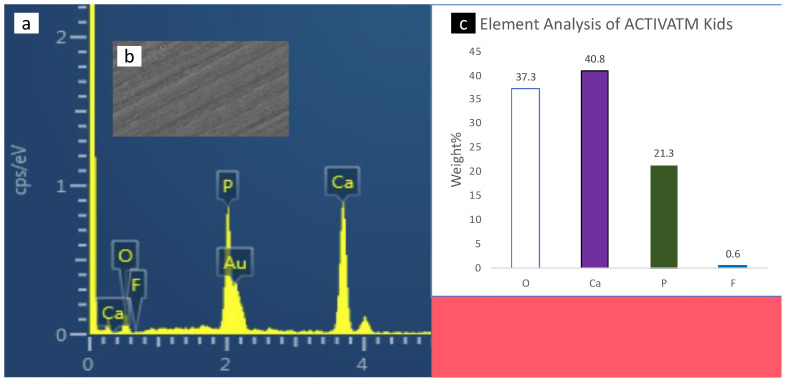
The SEM-EDX evaluation of the enamel samples under ACTIVA KIDS: (**a**) the SEM-EDX spectrum shows the dominant elements; (**b**) SEM image of the area for which the EDX spectrum was acquired; (**c**) concentrations of elements in weight (weight percent) of the oxygen, calcium, phosphate and fluoride elements found in enamel surface under the sealants.

**Figure 8:**
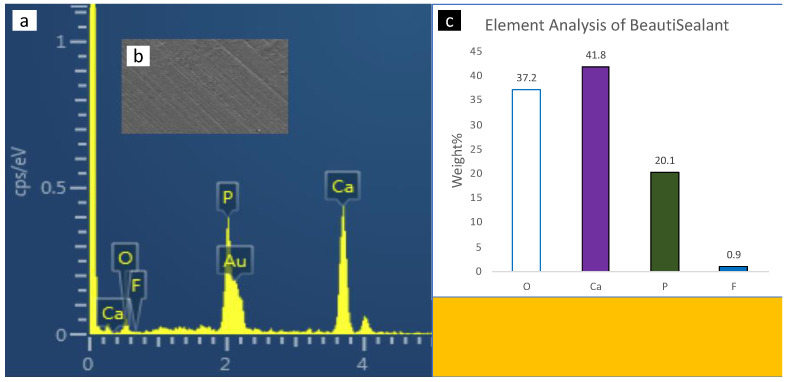
The SEM-EDX evaluation of the enamel samples under BeautiSealant: (**a**) the SEM-EDX spectrum shows the dominant elements; (**b**) SEM image of the area for which the EDX spectrum was acquired; (**c**) concentrations of elements in weight (weight percent) of the oxygen, calcium, phosphate and fluoride elements found in enamel surface under the sealants.

SEM-EDX spectroscopy of the surface enamel under the studies sealants and negative control are presented in Figure 4, Figure 5, Figure 6, Figure 7 and Figure 8. The main elements observed in the analysis of surface enamel were calcium, phosphorous and oxygen. Ions such as fluoride, silica and carbon were observed in minor quantities, where only fluoride was considered in addition to the main element in this study. Percentages of oxygen, calcium, phosphate and fluoride ions present in the surface enamel under the sealants are expressed in weight percentage. Bc and Bu showed higher Ca concentrations in comparison to the other groups.

### 3.4. Polarized Light Photomicrographs

**Figure 9:**
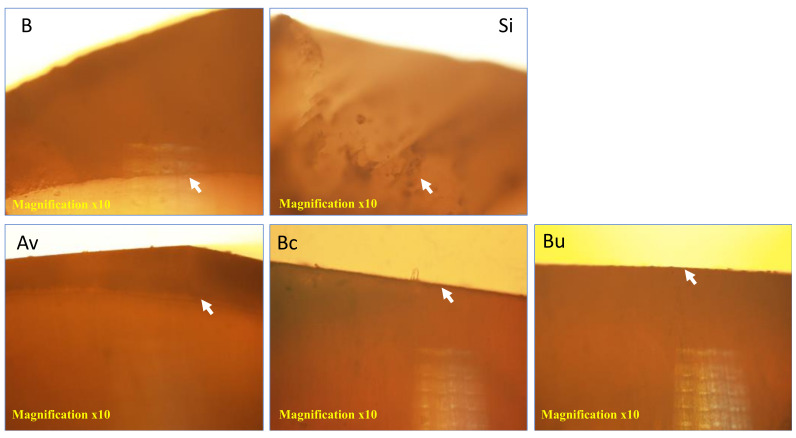
Photomicrographs of a longitudinal section of enamel under the sealed areas taken with polarized light. Dark bands at the surface (arrows) represent the demineralized enamel. (B) Blank and (Si) Seal-it sealant represent images under the negative control and non-bioactive sealant, respectively. (Av) ACTIVA KIDS Bioactive—restorative, (Bc) BioCoat^®^ sealant and (Bu) BeautiSealant show images of enamel under the bioactive sealants.

Figure 9 represents the polarized light photomicrographs of longitudinal sections of the samples after 7 days in the pH-cycling model. The dark bands on the surfaces represent the demineralized areas of the enamel. The negative control group and non-bioactive sealant showed wide dark bands that represent the demineralization areas. Av showed a wider demineralization band in comparison to the other bioactive groups. However, the demineralization band was narrower than both control groups. Bc showed a narrow demineralization band whereas Bu showed almost no demineralization on the surface of enamel.

## 4. Discussion

Pit and fissure sealants are one of the effective methods used for caries prevention [9]. However, cariogenic bacteria may still adhere to the sealant’s surfaces. The acids produced by the bacterial biofilm demineralize the tooth and initiate caries formation [2]. Thus, key strategies for preventing initial or secondary caries include stopping the demineralization cycle and bacterial adhesion to teeth surfaces, which conventional non-bioactive pit and fissure sealants may not be able to perform. Currently, enamel remineralization has been stimulated in the presence of bioactive pit and fissure sealants. Their tendency to release ions helps in the prevention of caries progression [24,25]. To address this issue, new preventative bioactive dental sealants that can suppress caries initiation or progression should be evaluated. Three bioactive sealants or materials that were recently introduced to the dental market were evaluated in comparison to two control groups (no sealant group and non-bioactive sealant).

Flexural strength has been used to assess the failure stress of dental materials while bending. It is considered more sensitive to minor changes in the structure compared to the compressive strength [41]. Pit and fissure sealants experienced mechanical stresses when applied on areas susceptible to mastication forces, thus sealants must have adequate mechanical qualities in addition to the remineralization abilities [42]. This study compared the flexural strength and elastic modulus of different pit and fissure sealants. The results indicated that Bc and Si showed higher mechanical strengths compared to Av and Bu. Moreover, flexural strength and elastic modulus of Bc showed significantly higher mechanical strengths in comparison to Av and Bu. Bu showed the lowest elastic modulus and significantly lower than the rest of the bioactive sealants. Av group had lower flexural strength in comparison to the flexural strength that was reported by the manufacturer [43]. However, the elastic modulus was found to be almost similar to the one reported by the manufacturer [43]. According to the ISO 4049, polymer-based restorative materials for occlusal surfaces must show a minimum flexural strength of 80 MPa [37]. All the evaluated sealants in this study showed higher values that are above the minimum required level. Previous laboratory studies revealed lower values of flexural strength of bioactive glass ionomer-based pit and fissure sealants compared to non-bioactive sealants [24,28]. Another study reported an almost similar range of flexural strength of experimentally developed bioactive sealants [21].

Mineral loss is considered one of the essential steps in the progression of enamel demineralization [27]. Studies suggested that bioactive materials may help in neutralizing the enamel surface demineralization effect of the bacterial acids by minerals gain [24,44,45]. To assess this effect, various demineralization–remineralization models have been used in in vitro or research laboratory settings. These models can be classified into microbial models and chemical models. In this study, we used a chemical model which has the advantage of simplicity, low cost, efficiency and stability of the experiment [46]. It was also shown that the use of lactic acid for 7 days at pH 5.4 had been proven to make a histological, clinical and radiographic deformation mimicking that of an early enamel lesion [47]. In this study, sealants were applied on sound enamel surface, then samples were alternated for 7 days between demineralization and remineralization solutions mimicking the oral environment where the pH is changing continuously. After the pH-cycling, the samples were assessed using a microhardness test, then SEM-EDX analysis and polarized imaging were done as complementary tests to measure the ions on enamel surface or the demineralization band thickness. Other assessment techniques for direct teeth mineral gain or loss assessments such as microradiography, cross-sectioned microhardness or indirect assessments such as iodide permeability, porosity or light-scattering could be used in future studies [48].

The results of this study revealed that bioactive pit and fissure sealants showed less surface hardness loss after 7 days of pH-cycling compared to the non-bioactive groups. Bc and Bu specifically promoted enamel remineralization and showed surface hardness gain. The mineral gain is linked to the compositions of these materials (Table 1). Calcium- and phosphate-containing materials provide more minerals and penetrate more in the enamel lesions compared to fluoride-releasing materials [49]. Bu contains surface pre-reacted glass–ionomer filler, which is known for its bioactive properties by releasing fluoride, sodium, strontium, aluminum, silicate and borate [50]. In addition, Bc contains microcapsules in its formulation which ensure controlled ion release and recharge [51].

This study supported the finding of previous studies which revealed that experimentally developed bioactive pit and fissure sealants could resist the demineralization effect of bacterial acids and/or demineralizing solutions compared to non-bioactive sealants [27,36].

The SEM-EDX analysis showed higher calcium and phosphate depositions for samples exposed to bioactive sealants, mainly Bc and Bu in comparison to the control groups. This could also be explained by the compositions of these sealants (Table 1). Corroborating with the quantitative outcomes from the microhardness testing and ions analysis, the polarized light photomicrographs confirm the differences between the control groups and bioactive sealants groups. The acids in contact with the enamel during the pH-cycling could create disorganization of prisms and a change in the birefringence. Enamel backscatter can be affected by mineral loss [52,53]. A thinner demineralized zone was seen in the images when the enamel is treated with the bioactive sealants, mainly Bc and Bu. This suggests that there was a higher demineralization process that occurred in the control groups in comparison to the bioactive sealants.

Overall, the evaluated bioactive materials in this study showed excellent performance in the assessed properties. Further assessments, such as the amount of ions released and their ability to be re-charged, are recommended to be conducted in future studies. Moreover, the clinical assessments of these new bioactive sealants in terms of retention and caries prevention are highly suggested. Although this in vitro study was able to assess some of the remineralization and mechanical properties of the studied materials, it has some limitations, such as the short-term assessments and that no quantitative measurements of the ions released into the deep enamel layer were performed.

## 5. Conclusions

In this study, Bc sealant showed higher remineralization abilities than the other sealants after 7 days of pH-cycling, as well as higher flexural strength and elastic modulus. Bc and Bu sealants showed the highest percentage of calcium, phosphate and fluoride ions and the thinnest demineralization band. These new bioactive resin-based sealants seem to have promising remineralization abilities that could overcome some of the clinical drawbacks of conventional non-bioactive sealants.

## Figures and Tables

**Table 1:** Studied materials.

Name	Type	Composition	Manufacturer	Code
Blank	Negative control	-	-	B
Seal-it sealant	Non-bioactive sealant	Bisphenol A ethoxylate dimethacrylate of isomers (30–50%) Triethylene glycol dimethacrylate (20–30%) Camphorquinone (<0.1%)	SPIDENT, Incheon, South Korea	Si
ACTIVA KIDS Bioactive—restorative	Bioactive material	Mix of methacrylates and diurethane with modified polyacrylic acid (44.6%), silica, amorphous (6.7%), sodium fluoride (0.75%)	Pulpdent, Watertown, MA, USA	Av
BioCoat® sealant	Bioactive sealant	Triethylene glycol dimethacrylates, (1-methylethylidene)bis[4,1-phenyleneoxy(2-hydroxy-3,1 propanediyl)] bismethacrylate, fumed silica, barrium aluminoborosilicate (<60%); calcium donor (<2%); photo-initiator (<2.5)	Premier, Plymouth Meeting, PA, USA	Bc
BeautiSealant	Bioactive sealant	Primer: acetone, phosphoric acid monomer, carboxylic acid monomer, distilled water. Sealant: S-PRG fillers (30% by weight), micro fumed silica, UDMA, TEGDMA.	SHOFU, Kyoto, Japan	Bu

**Table 2:** Means ± SD of flexural strength, elastic modulus and surface hardness change (%).

Material Type	Flexural Strength Mean ± SD (MPa)	Elastic Modulus Mean ± SD (GPa)	Surface Hardness Change Mean ± SD (%)
Negative Control	Not applicable	Not applicable	−70.1 ± 3.4 ^b^
Si	84.7 ± 6.6 ^ab^	4.6 ± 0.4 ^ab^	−54.9 ± 7.5 ^b^
Bc	94.4 ± 9.7 ^a^	5.2 ± 0.5 ^a^	52.9 ± 24.8 ^a^
Av	79.9 ± 6.9 ^b^	4.4 ± 0.4 ^bc^	−35.1 ± 7.4 ^b^
Bu	78.4 ± 9.2 ^b^	3.9 ± 0.6 ^c^	39.7 ± 18.4 ^a^

Values indicated by different letters (a, b, c) are statistically significant and different from each other (*p* < 0.05).

## Data Availability

Data are available upon request from the corresponding author.
